# iGenSig-Rx: an integral genomic signature based white-box tool for modeling cancer therapeutic responses using multi-omics data

**DOI:** 10.21203/rs.3.rs-3649238/v1

**Published:** 2023-11-30

**Authors:** Sanghoon Lee, Min Sun, Yiheng Hu, Yue Wang, Md N. Islam, David Goerlitz, Peter C. Lucas, Adrian V. Lee, Sandra M. Swain, Gong Tang, Xiao-Song Wang

**Affiliations:** University of Pittsburgh; University of Pittsburgh; University of Pittsburgh; University of Pittsburgh; Georgetown University Medical Center; Georgetown University Medical Center; University of Pittsburgh; University of Pittsburgh; National Surgical Adjuvant Breast and Bowel Project (NSABP); University of Pittsburgh; University of Pittsburgh

## Abstract

Multi-omics sequencing is expected to become clinically routine within the next decade and transform clinical care. However, there is a paucity of viable and interpretable genome-wide modeling methods that can facilitate rational selection of patients for tailored intervention. Here we develop an integral genomic signature-based method called iGenSig-Rx as a white-box tool for modeling therapeutic response based on clinical trial datasets with improved cross-dataset applicability and tolerance to sequencing bias. This method leverages high-dimensional redundant genomic features to address the challenges of cross-dataset modeling, a concept similar to the use of redundant steel rods to reinforce the pillars of a building. Using genomic datasets for HER2 targeted therapies, the iGenSig-Rx model demonstrates stable predictive power across four independent clinical trials. More importantly, the iGenSig-Rx model offers the level of transparency much needed for clinical application, allowing for clear explanations as to how the predictions are produced, how the features contribute to the prediction, and what are the key underlying pathways. We expect that iGenSig-Rx as a class of biologically interpretable multi-omics modeling methods will have broad applications in big-data based precision oncology. The R package is available: https://github.com/wangxlab/iGenSig-Rx.

**NOTE: the Github website will be released upon publication and the R package is available for review through google drive**: https://drive.google.com/drive/folders/1KgecmUoon9-h2Dg1rPCyEGFPOp28Ols3?usp=sharing

## BACKGROUND

With the advent of low-cost genome sequencing, precision oncology is expected to undergo a deep transformation by leveraging multi-omics sequencing to guide precision treatment decisions, which is deemed to be highly cost-effective. While there is copious literature on the topic of predicting treatment responses based on multi-omics data in cancer, most, if not all, available modeling tools for precision oncology are machine-learning based tools that lack the transparency much needed for clinical use. To date there is a serious lack of white-box methods for modeling therapeutic responses based on clinical trials. In addition, most of these tools are developed for modeling pharmacogenomics data of cancer cell lines and are not validated for directly modeling clinical trials [1–6]. Whereas most of the clinical trial studies used standard machine learning methods for modeling therapeutic responses [7–11], rather than specialized tools. This reflects a dearth of specialized multi-omics modeling tools for clinical trials.

More importantly, machine learning methods are predominantly developed from image and language processing, which lack specialized algorithms in design to deal with the hallmark characteristics of genomic data: i) genomic data have ultra-high dimension with massive numbers of genomic features, ii) clinical trial datasets typically have very limited subjects, iii) high multicollinearity of genomic features resulting from co-expression and co-occurring genetic events, iv) genomic data are imprecise and inconsistent as a result of sequencing errors, experimental variations, library preparation methods and platforms, discordant sequencing depth and read-length, heterogenous sample qualities. This problem is further exacerbated by the black-box nature of the machine learning algorithms that lack the transparency much needed for clinical use, which raises concerns from oncologists and questions from patients. These factors create an urgent need for innovative white-box methods designed from scratch that are specially adapted to the hallmark characteristics of genomics data and are more suitable for clinical applications that require high-levels of transparency.

While many effective therapies have been developed for breast cancer, overtreatment of clinically localized or regional tumors remains a major clinical problem. For example, compelling evidence suggests that HER2 targeted therapy is highly effective in the treatment of HER2-positive breast cancer, but the responses are discordant, leading to overuse of HER2 monoclonal antibodies in non-responders which carry more risks than benefits. The lack of genomic signatures underlying the differential clinical response to HER2-targeted therapy has a negative impact on the development of cost-efficient strategies for better management of HER2-positive breast cancer. Recently, clinical trials in HER2-positive breast cancer patients with Trastuzumab-based neoadjuvant chemotherapy have produced a tremendous amount of multi-omics data along with well documented therapeutic endpoints, which provided great opportunity to develop big-data based predictive models [7, 8, 12–15].

In our previous study, we postulated that the collinearity of high-dimensional features may actually help improve the cross-dataset applicability of predictive models, similar to the use of redundant steel rods to reinforce the pillars of a building. We thus developed a new line of modeling methods that generates prediction scores using high-dimensional redundant genomic features predictive of therapeutic responses detected from labeled genomic datasets, then reduce the effect of feature redundancy via adaptively penalizing the collinearity of predictive features in specific tumors based on unlabeled datasets for large tumor cohorts [16]. With this approach, if a subset of genomic features was lost due to sequencing noise or experimental variations, the redundant features will sustain the predictive power of the model. The unbiased genomic information acquired from large cancer cohorts will substantially improve the transferability of the models to clinical study of heterogenous patient cohorts. iGenSig modeling diminishes false positives resulting from sequencing errors and overweighing via averaging the weights of genomic features and prevents overfitting via dynamically adjusting the feature weights for training subjects. Furthermore, we have demonstrated the general applicability of our iGenSig methods to model targeted therapy and chemotherapy in a variety of cancer types based genomic datasets for chemical perturbations, and we have validated our models for five different treatments on six clinical trial datasets [16].

In this study, we aim to further develop this technology into a white-box tool called iGenSig-Rx for modeling pathological responses, which is in high demand for genomic study of clinical trials. In contrast to our previous method, modeling the binary pathological response is more challenging than modeling the continuous drug sensitivity measurements due to the lack of information about the precise degrees of responses. To develop the modeling method, we focused our study on modeling HER2 targeted therapy in breast cancer for which multiple clinical trial datasets with relatively large patient numbers are available to test the transferability of the model. The benefit of using clinical trial datasets lies in that the pathological responses as clinical endpoint are directly associated with the specific treatment. Whereas retrospective clinical studies are inconsistent in treatment regimens, and the outcome are more likely to be confounded by sequential treatments the patient received. Our results showed that the iGenSig-Rx model developed in this study demonstrates stable predictive power across four independent clinical trials and reveals clinically relevant insights into the pathways underlying HER2 therapy responses. Taken together, iGenSig-Rx is designed to address transparency, cross-dataset applicability, and interpretability issues for big-data based modeling, with specialized algorithms adapted to the hallmark characteristics of genomic data, thus will have broad applications in big-data based precision oncology.

## METHODS

### The basic algorithm to calculate genomic feature weight and select significant genomic features

**Figure 1:**
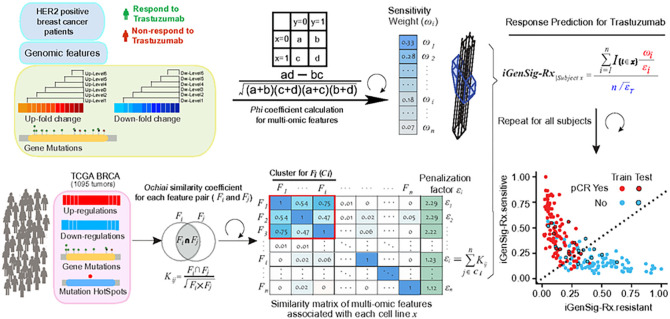
The principle and algorithm design of integral genomic signature analysis for predicting therapeutic response (iGenSig-Rx) in cancer. The upper panel shows the calculation of the weights for significant genomic features by using Phi Correlation. The weights represent the strength of association of genomic features with drug sensitive or resistant tumors and are used to calculate iGenSig-Rx sensitive or resistant scores of patient subjects. The lower panel shows the computation of a similarity matrix for genomic features based on TCGA Pan-Cancer dataset to penalize the redundancy between the genomic features associated with each patient *x.* The resulting penalization factors are formulated to calculate iGenSig-Rx sensitive or resistant scores, as depicted in the formula on the top right. In the dot plot of iGenSig-Rx sensitive and resistant scores for all patients, the best distinction line (D-line) for separating true sensitive from resistant patients is calculated. The distance of each patient to this D-line is defined as the “iGenSig-Rx” score. Positive and negative iGenSig-Rx values indicate sensitive and resistant response predictions, respectively.

The workflow of the iGenSig-Rx model is depicted in Fig. 1. To define the weight $\left(\omega_{i}\right)$ of each genomic feature in sensitive or resistant therapeutic response, we leveraged the Phi coefficient, also called a mean square contingency coefficient, to calculate the association between individual genomic features and therapeutic sensitive or resistant patients.


1) $${Sensitivity\;Weight,w}_{i}=Pearsoncoefficientbetweenith\;genomic\;feature\;and\;therapeutic\;sensitive\;patients$$

The association will be represented as the enrichment of therapeutic sensitive or resistant patients in each genomic feature. We assessed the observed enrichment by removing genotypes with a Phi coefficient < 0.13, which shows the best performance in the drug response prediction. To eliminate potential bias, we removed genomic features that belong to ‘Up_Level1’ and ‘Down_Level1’, because the effect of low-level genomic features is feeble. Likewise, we removed gene expression features that show same trend of predictive values on pCR when the genes are either up or down regulated.

To prevent the inflation of iGenSig-Rx scores by genomic feature redundancy, we leveraged the TCGA Pan-Cancer RNA-seq and exome datasets to assess the co-occurrence between genomic features associated with each patient. We generated the similarity matrix of genomic features based on the Otsuka-Ochiai coefficient between the genomic features. We defined $K_{ij}$ for the Otsuka-Ochiai coefficient between the pair of i th and the j th genomic feature associated with patient x. We then introduced a penalization factor (ε) for the ith genomic feature as the sum of the coefficients obtained from the similarity matrix of genomic features associated with a given patient x

2) $$\epsilon_{i}=\sum_{j=1}^{n}\;K_{ij}$$

where n is the total number of genotypes associated with a x. We then eliminated the cumulative effect of nonsignificant overlaps between genomic features. To achieve this, we made clusters of genomic features by hierarchical clustering analysis with the ‘complete’ agglomeration method and clustering height=2 and excluded the coefficients of genomic features from outside the cluster. Here $\omega_{i}$ is an estimator of redundancy among the genomic features associated with a patient x.

### Calculate iGenSig-Rx scores for predicting therapeutic responses

We then penalized the weight $\omega_{i}$ using the square root of $\varepsilon_{i}$ resulting in Effective Weight (EW):

3) $$EW_{i}=\frac{\omega_{i}}{\epsilon_{i}}$$

The sum of the reciprocals of square root $\varepsilon_{i}$ was then used to calculate the Effective Feature Number (EFN):

4) $$EFN_{i}=\sum_{i=1}^{n}\;\frac{1}{{\overset{‾}{\epsilon }}_{T}}$$

Finally, the iGenSig-Rx score of the given patient x is computed as:

5) $$iGenSig-oncologist\;{\;}_{|{\mathrm{patient}\;}_{x}}=\frac{\sum_{i=1}^{n}EW_{i}}{EFN_{i}}=\frac{\sum_{i=1}^{n}I_{\left\{ix\right\}}\frac{\omega_{i}}{\epsilon_{i}}}{{\overset{‾}{\epsilon }}_{T}}$$

The slope of the dividing line (D-line) for sensitive and resistant patients is determined by Youden Index. We then calculated the distance between a patient and D-line and defined the distance as the final iGenSig-Rx score, which can be used to predict the patient’s treatment sensitivity. The sensitive patients above D-line will have positive iGenSig-Rx scores and vice versa.

The methods for retrieval of clinical trial datasets, multi-omics feature extraction, determining the D-line, feature error simulations, benchmarking, machine learning, pathway interpretation, and statistical analysis are provided in **Supplementary Methods.**

## RESULTS

### Modeling patient responses to trastuzumab and paclitaxel-based chemotherapy

To build the iGenSig-Rx predictive model for standard HER2 targeted therapy and chemotherapy, we analyzed multi-omics data from the treatment arms of the CALGB 40601 trial testing Trastuzumab in combination with paclitaxel, with or without Lapatinib in HER2-positive patients **(Table 1)**. Differential expression features representing twelve levels of up- or down-regulated genes were extracted from the RNA-seq data for both trials (Fig. 1). In addition, we generated mutation features based on a total of 19,288 somatic nonsynonymous mutations and 794 adjacent gene rearrangements (AGRs) in the CALGB 40601 cohort (**Supplementary Tables 1 and 2**). We obtained 6,685 somatic mutations in the ACOSOG Z1041 cohort provided by the respective publications [8] and generated 33,152 AGRs (**Supplementary Table 3**). The mutation features are then integrated with differential expression features representing twelve levels of up- or down-regulated genes.

**Figure 2:**
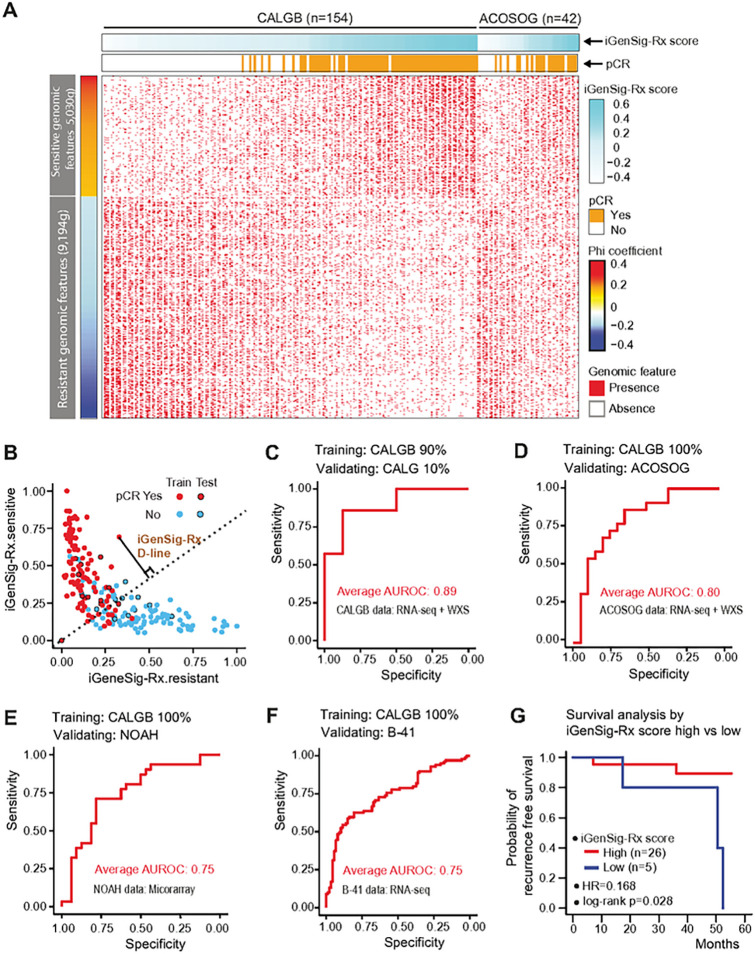
The performance of iGenSig-Rx models in predicting the treatment responses in training and validation clinical trial datasets for HER2 targeted therapies in breast cancer (A) The association between significant genomic features, pCR rate, and iGenSig-Rx scores. The enrichment of drug-resistant and sensitive significant genomic features (n=3,955) based on Phi correlation is shown in the figure. The CALGB 40601 subjects are sorted by their iGenSig-Rx scores in column (light blue bars on the top). (B) iGenSig-Rx sensitive and resistant scores for CALGB 40601 subjects. Red dots represent pCR-achieved subjects, and blue dots present non-pCR-achieved subjects. Black circles indicate the subjects used in the test set. (C) Area under the receiver operating characteristic (AUROC) curve displays the performance of the predicting sensitive responses to Trastuzumab-based treatment in CALGB 40601. 90% of subjects of CALGB 40610 were used as the train set, and 10% of subjects were used as the test set. (D-F) The performance of the predicting sensitive responses in ACOSOG Z1041, NOAH, or NSABP B-41 as the test set. 100% subjects of CALGB 40601 were used as the train set. (G) Kaplan-Meier plots show the predictive values of the iGenSig-Rx model on ACOSOG Z1041.

We then selected the predictive genomic features based on their correlations with pathological responses computed using phi correlation coefficients. Figure 2A shows the heatmap of significant genomic features correlating with iGenSig-Rx scores and pCR in the CALGB 40601 and ACOSOG Z1041 trials. Next, we integrated a TCGA gene expression profile and somatic mutation datasets of 1,095 breast tumors to quantify the similarity between genomic features associated with each tumor in the clinical trials and applied the measurement of the similarity to the redundancy penalty score in individual genomic features. To develop the iGenSig-Rx model, we made a random sampling that select 90% of subjects in CALGB 40601 trainset and the rest 10% as the internal test set. We then calculated the iGenSig scores predicting the sensitive or resistant responses for each subject based on the correlated genomic features (Fig. 2B). The final iGenSig-Rx scores are calculated based on the distance of each subject to the division line (D-line) that best separates the responders from non-responders (Fig. 2B). The iGenSig-Rx scores are positively correlated with the pCR-achieved subjects with a similar trend in both training and testing sets as exemplified in the CALGB 40601 model. The iGenSig-Rx scores calculated for each subject can be used to predict Trastuzumab and Paclitaxel based therapeutic response.

### The predictive performance of the iGenSig-Rx model

We optimized the iGenSig-Rx model by tuning the parameters such as the cut-off for selecting predictive genomic features for iGenSig-Rx modeling and the formula for calculating iGenSig-Rx scores. We built the models based on ten random samples of the trainsets in the CALGB 40601 dataset, and calculated Area Under ROC Curve (AUROC) based on pCR in test sets to assess the model’s prediction performance. The iGenSig-Rx model predicted therapeutic response on CALGB 40601 subjects with an average of AUROC 0.91 in trainsets and 0.89 in internal test sets based on 10 permutated training and testing sets (Fig. 2C).

Next, we sought to examine the value of the iGenSig predictive model in independent clinical trials for trastuzumab and paclitaxel-based regimens. To achieve this, we accessed genomic datasets for two large clinical trials. NOAH (NeOAdjuvant Herceptin) is an open label phase 3 trial evaluating neoadjuvant doxorubicin/paclitaxel (AT) followed by cyclophosphamide + methotrexate+ fluorouracil (CMF) in combination with trastuzumab in breast cancer patients [17]. The NOAH clinical trial only have microarray gene expression profile data [18], and the arm3 subjects (n = 63) (HER2-positive, Trastuzumab-treated) were included in our analysis. The NSABP B-41 trial is a Randomized Neoadjuvant Trial for HER2-positive operable breast cancer treated with neoadjuvant trastuzumab and chemotherapy (AC + T), with or without lapatinib [19]. Transcriptome sequencing data on the pretreatment tumors are available for NSABP B41 trial for approximately 250 patient subjects with well documented clinical and treatment outcome information. Since both trials lack genomic sequencing data, we generated genomic features based on transcriptomics only. We benchmarked the models to the three external validation sets, ACOSOG Z1041, NOAH, and NSABP B41 to assess the cross-dataset performance of the iGenSig-Rx model. As expected, the performance of our iGenSig models achieved higher AUROC of 0.80 in the ACOSOG Z1041 trial that has both gene expression and whole exome sequencing (WXS) data, and 0.75 in both NOAH and NSABP B41 trials that has only transcriptomic data (Fig. 2D, E, and F). When we removed genomic features by somatic mutations in CALGB 40610 training set and ACOSOZ Z1048 validation set, the iGenSig-Rx mode performance was worse (AUROC of 0.77 in blue in Fig. 2D). In addition, the iGenSig-Rx model successfully predicted recurrence-free survival in the ACOSOG trial, the only dataset that has recurrence free survival data. The favorable survival in ACOSG with a hazard ratio of 0.168 and log-rank p-value of 0.028 (Fig. 2G).

### The iGenSig-Rx model does not depend on the genomic features of drug target genes or hormone receptor genes

**Figure 3:**
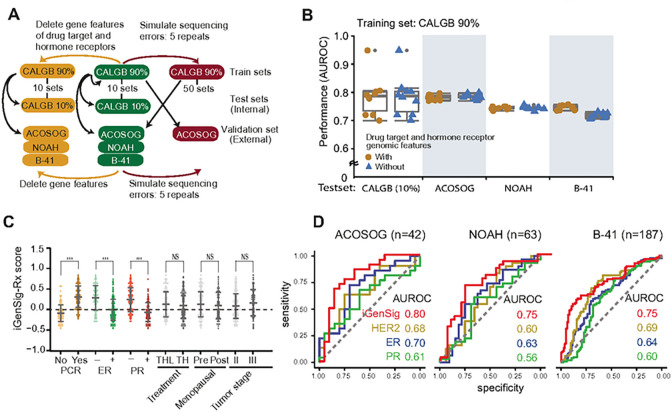
The iGenSig-Rx scores showed better predictive values compared to HER2 and hormone receptors and is not dependent on genomic features derived from these receptor genes. (**A**) Schematic diagram to represent the train, test, and validation sets used to investigate the resilience of the iGenSig-Rx model against devoid of hormone receptor genes (Fig. 3B) and simulated sequencing errors in genomic features (Fig. 4D). (B) The performance of the iGenSig-Rx model is not dependent on genomic features derived from HER2 and hormone receptor genes. The prediction model’s performance was compared between all genomic features and the genomic features devoid of HER2 and hormone receptor genes. (C) The iGenSig-Rx scores are associated with pCR achievement, ER, and PR subtypes. However, the scores are not associated with treatment arms, menopausal, or tumor stages. THL, Paclitaxel (T) + Trastuzumab (H) + Lapatinib (L); TH, Paclitaxel (T) + Trastuzumab (H). (D) The comparison of prediction performance AUROCs between iGenSig-Rx and HER2, ER, and PR expression levels in ACOSOG Z1041, NOAH, or NSABP B-41 trials.

To examine the dependency of iGenSig-Rx predictions on the genomic features of the primary drug target and hormone receptor genes, we depleted the genomic features of ERBB2 or ESR1 genes from the whole genomic features in the CALGB 40601, ACOSOG Z1041, NOAH, and NSABP B-41 datasets (Fig. 3A). Our results showed that the performance of the iGenSig-Rx model is not affected by the absence of genomic features of known Trastuzumab target and hormone receptor genes (Fig. 3B).

### The association of iGenSig-Rx scores with clinicopathological variables

Next, we examined the association of iGenSig-Rx scores with the pathological responses of patient subjects stratified based on receptor status and clinicopathological subtypes. The median level of iGenSig-Rx scores was significantly higher in subjects achieving pCR but lower in ER-positive and PR positive subjects in the CALGB 40601 trial (Fig. 3C). This suggests that the iGenSig-Rx scores are positively associated with pCR rate but negatively correlated with ER and PR subtypes. The iGenSig-Rx scores did not show associations with treatment arms, menopausal status or tumor stage, which is consistent with the results of the CALGB 40601 clinical trial [7] that the treatment arms did not affect the patient outcome.

Previous studies reported that high levels of HER2 expression and low ER levels are associated with increased benefit of Trastuzumab-based therapy [9, 20]. We thus examined the correlation between the iGenSig-Rx scores and the ER, PR, and HER2 gene expression levels in the CALGB 40601 dataset. Our results show that the iGenSig-Rx scores are positively correlated with HER2 expression (R = 0.556, p < 0.001) but negatively correlated with ER (R=−0.554, p < 0.001) and PR expression (R=−0.496, p < 0.001) **(Supplementary Fig. 1)**. This suggests that the HER2, ER, and PR pathway signatures may have major contributions to the iGenSig model. However, the iGenSig model do not rely on the genomic features from these receptor genes as it is grounded on the integral signature of all genomic features associated with these receptor genes. Next, we compared the prediction performance between iGenSig-Rx scores and the expression of known biomarkers HER2, ER, and PR in the ACOSOG Z1041, NOAH, and NSABP B-41 trial datasets. Our result showed that the iGenSig-Rx model outperformed the biomarker expressions on predicting the pCR of all three trials (Fig. 3D).

### Comparison of the predictive performance between the iGenSig-Rx model and standard machine learning models in the presence or absence of simulated errors in genomic features

**Figure 4:**
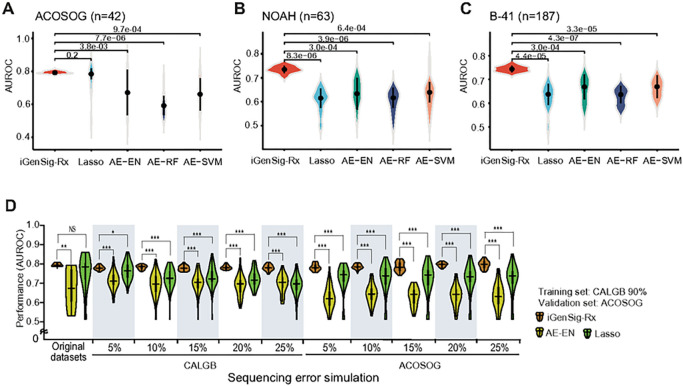
Comparison of the iGenSig-Rx models with standard machine learning models on predicting the response of HER2 positive breast cancer patients to trastuzumab and paclitaxel-based regimen. (A, B, and C) Comparison of prediction performance between iGenSig-Rx model and Al-based methods on CALGB 40601 train and ACOSOG Z1041, NOAH, and B-41 validation set. For Al-based methods, the unsupervised learning was performed by autoencoder (AE) and supervised learning was performed using various machine learning tools, including Lasso, elastic net (EN), random forest (RF) and support vector machine (SVM). (D) The prediction performance of iGenSig-Rx, Al-based method AE-EN, and LASSO on CALGB and ACOSOG trials under simulated sequencing error rates. The 90% subjects of CALGB 40601 are randomly selected as train set, and the subjects from ACOSOG Z1041 trial are used as validation set. * P<0.05, ** P<0.01, and *** P<0.001 (unpaired two-tail t-test).

Next, we sought to compare the performance of iGenSig-Rx modeling with the AI- and machine learning-based approaches implemented in other studies [1,2, 7, 21]. Following the previous reports [1,22] for dimensionality reduction, we computed the unsupervised representation of the genomic features based on the autoencoder deep learning method. Then, the dimensionality reduced data were used in the machine learning methods, such as elastic net, random forest (RF), or support vector machine (SVM), for supervised learning on drug responses. In addition, we also performed modeling directly from the high-dimensional genomic features using the minor absolute shrinkage and selection operator (Lasso), one of the few standard machine-learning methods that can deal with massive number of genomic features with high multicollinearity [23]. Compared to the iGenSig-Rx model, Lasso achieved the prediction performance AUROC 0.69 ~ 0.83 (median 0.79), and the Al-based methods achieved AUROC 0.5 ~ 0.8 (median 0.68) on ACOSOG Z1041, NOAH, and NSABP B-41 data (Fig. 4A–C). These AUROCs Al-based methods achieved are much lower than the one iGenSig-Rx model achieved, 0.79 ~ 0.81 (median 0.80). When applied to NOAH and NSABP B-41 validation set, the AUROCs of prediction for these methods dropped to a median range of 0.50 ~ 0.68 or 0.58 ~ 0.73, respectively. In contrast, the iGenSig-Rx models maintained significantly higher predictive values (Fig. 4A–C). In addition, the range of AUROC values by Al-based methods showed wide variations.

To assess the resilience of the iGenSig-Rx model against the common genotypic bias that can be caused by insufficient depth or sequencing or misreading gene expression, we simulated the errors in genomic features with 5–25% rates by randomly generating false-positive or false-negative genomic features in either CALGB 40601 or ACOSOG Z1041 dataset (Fig. 3C; see Methods). We built the iGenSig-Rx and Al-based method models using the genomic features containing simulated errors for comparison. The result showed that the predictive performance in the autoencoder-elastic net (AE-EN) or Lasso model was substantially destabilized even on 5% of simulated genotypic errors, and it got worse as the error rate increased (Fig. 4D). In contrast, the iGenSig-Rx models can tolerate the simulated errors in genomic features for up to 25% without a significant decrease in their performance, regardless of if the genotypic errors are generated in training or validation sets.

### Clinical variables that confound the iGenSig-Rx model

**Figure 5:**
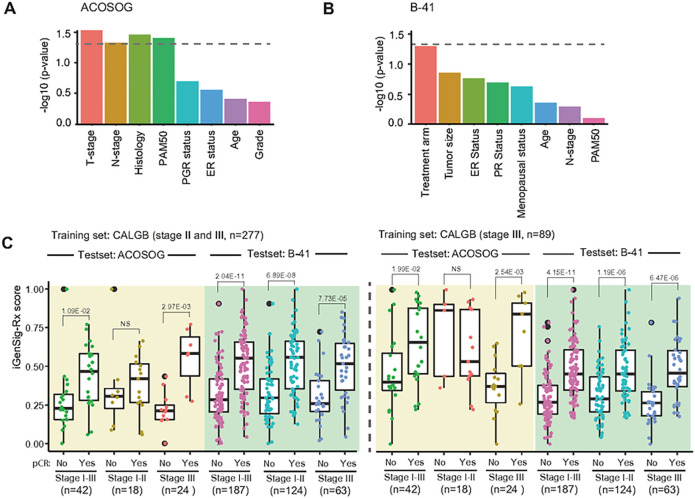
The confounding clinical variables to the iGenSig-Rx prediction model. (A-B) The interactions of confounding clinical variables were assessed based on multiple logistic regression models for therapeutic response predictive values in ACOSOG Z1041 and NSABP B-41. The bar plot shows the −log10 transformed p values of Chi-Square tests comparing the pair-wise multiple logistic regression models with the simple logistic regression model. (C) The predictive values of the iGenSig-Rx model are displayed for ACOSOG Z1041 and B-41 subjects stratified by tumor stages; stage I-III, stage I-II or stage III. The predictive values were measured in pCR or non-pCR subjects separately in each tumor stage stratification. CALGB 40601 stage II-III (n=277) or CALGB 40601 stage III (n=89) were used as the train sets to build the independent iGenSig-Rx modeling. ACOSOG Z1041 and NSABP B-41 are used as the test sets.

Next, we sought to examine if key clinical variables such as PAM50, tumor stage, grade, age, receptor status, etc., may confound the predictive effect of the iGenSig-Rx models. For example, HER2-positive tumors that express ER are known to respond well to endocrine therapy [24]. Age is one of the most critical risk factors for cancer progression [25]. The tumor microenvironment by different tumor stages are known to influence therapeutic response and clinical outcomes [26]. The interactions of the iGenSig-Rx model with the possible confounding variables were assessed using logistic regression (see Methods). Our result showed that among the clinical variables, tumor stage/tumor size appear to be most significantly interacting clinical variable in both datasets (Fig. 5A and B). We thus stratified the ACOSOG Z1041 and NSABP B-41 subjects into low stage (I-II) and high stage (III) subjects and calculated the predictive values (iGenSig-Rx scores) trained on all CALGB 40601 subjects (n = 277) or stage III subjects only (n = 89), respectively. Interestingly, stage Ill subgroups in ACOSOG Z1041 and NSABP-B41 test sets showed higher predictive values (lower p-values) when modeled based on stage Ill subgroups only in the CALGB 40601 trial compared to that modeled based on all subjects, particularly in the NSABP-B41 trial (Fig. 5C). This suggests that the therapeutic response prediction is associated with tumor stages and stratifying the patient subjects based on tumor stage may help improve the modeling outcome.

### The signature pathways underlying the integral genomic signature of HER2-targeted therapy response

**Figure 6:**
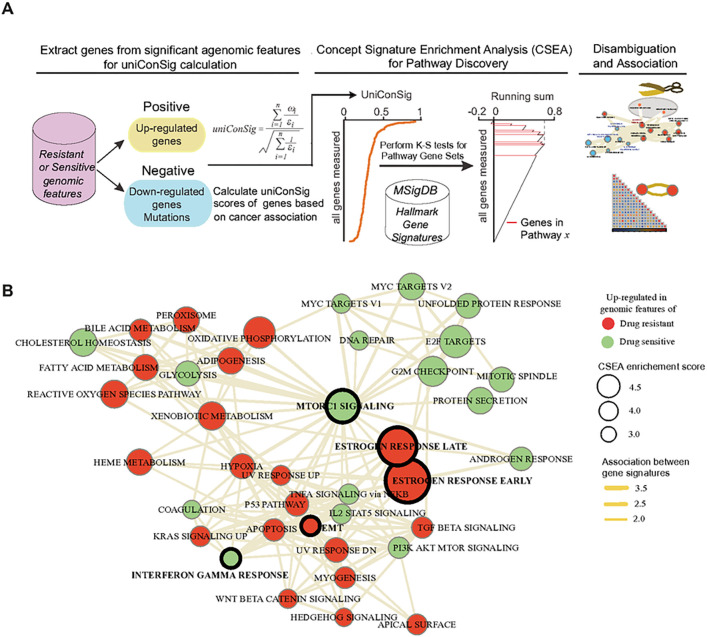
CSEA reveals the network of the signature pathways underlying the iGenSig-Rx model for trastuzumab and paclitaxel-based treatment response. (A) CSEA facilitates the interpretation of pathways underlying the iGenSig-Rx model. Using positive or negative contributing genes to the iGenSig-Rx model, a uniConSig score can be calculated for all human genes in the. Then the enrichment of pathways in this sorted gene list can be assessed by K-S tests. (B) The top up-regulated pathways predicting sensitive response (green) or resistant response (red) to trastuzumab and paclitaxel-based regimen were clustered in the interconnected network. The node’s size depicts the CSEA enrichment score for each pathway, and the thickness of the edge depicts the functional associations between the pathways computed based on CSEA (see Methods).

Next, we examined the signature pathways underlying the integral genomic signature of HER2-targeted therapy response based on the CSEA method [27] we developed (Fig. 6A). CSEA assesses functional enrichment of pathways in the signature gene list extracted from therapeutic sensitive or resistant genomic features. CSEA deep interprets the function of the signature gene list via computing their overrepresentations in a wide array of molecular concepts, which were then used as weights to compute a genome-wide uniConSig score that represent the functional relevance of human genes underlying this signature gene list. Then the UniConSig-sorted genome will be used for testing pathway enrichments. The most relevant up-regulated gene signatures predicting sensitive responses are MTOR1 signaling, MYC targets, and interferon gamma response (Fig. 6B, **Supplementary Fig. 2, and Supplementary Table 4)**. This is consistent with the fact that phosphatidylinositol-3 kinase/mechanistic target of rapamycin (PI3K/mTOR) signaling mediates HER2 downstream signaling and is implicated in the pathogenesis of HER2-overexpressing breast cancers. Among the up-regulated gene signatures predicting lack of treatment responses, estrogen response pathways and epithelial mesenchymal transition (EMT) pathways are of most interest (Fig. 6B, **Supplementary Fig. 2, Supplementary Table 5)**. Estrogen receptor (ER) is an established oncogene known to drive resistance to HER2-targeted therapy through ER-driven growth signaling independent of HER2[28]. EMT has been reported to mediate resistance to HER2, and EGFR inhibitors [29–31] and Paclitaxel, consistent with our previous study [32].

## DISCUSSION

In this study, we developed iGenSig-Rx modeling methods for modeling therapeutic responses based on clinical trial datasets grounded on the integral genomic signature analysis we developed for modeling chemical perturbations of cancer cell lines [16]. Our iGenSig-Rx methods implement innovative designs to address the five hallmark characteristics of cancer genomics data and provide robust clinical decision support with high transparency and cross-dataset applicability: i) This method leverages the high-dimensional redundant genomic features and introduces de novo redundant genomic features to enhance the transferability of multi-omics-based modeling for precision oncology, a concept like building constructions that use multiple steel rods to reinforce the pillars of a building. With this method, we speculate that if a subset of the genomic features was lost due to sequencing biases or experimental variations, the redundant genomic features will help sustain the prediction score. ii) To overcome the limited number of subjects, iGenSig detects the co-occurrence of genomic features using unlabeled genomic datasets for large cohorts of human cancers from The Cancer Genome Atlas (TCGA). This method also prevents overfitting through dynamically adjusting the feature weights for training subjects. iii) To address the multi-collinearity issue, the iGenSig algorithm adaptively penalizes the redundant features detected in specific samples, allowing for preservation of redundant genomic features during the modeling, while preventing the feature redundancy from flattening the scoring system. The second genomic information obtained from unlabeled large cancer cohorts will substantially improve cross dataset applicability of the iGenSig models, particularly on clinical trial datasets. iv) To deal with the imprecise nature of genomic data, iGenSig modeling utilizes the average correlation intensities of significant genomic features detected in specific samples to diminish the effect of false positive detection resulting from sequencing errors and overweighing. Thus iGenSig-Rx represents a new class of integral multi-omics modeling methods for big-data precision medicine.

To examine the utility of our iGenSig-Rx method in modeling clinical trial datasets, we utilized multiple genomic datasets of clinical trials for HER2 targeted therapy in breast cancer patients, including CALGB 40601, ACOSOG Z1041, NOAH, and NSABP B-41. The prediction AUROC of our iGenSig-Rx models achieved 75 ~ 0.8 in all three independent validation sets, ACOSOG Z1041, NOAH, and NSABP B-41. The flexibility of our iGenSig modeling in handling different types of omics data allow for integration of additional multi-omics features that could further improve the precision of the therapeutic response prediction. More important, grounded on the CSEA method we developed [33], the pathways underlying the iGenSig models are highly interpretable, which is one of the advantages of the iGenSig-Rx model over black box approaches. CSEA revealed the pathways characteristics of the integral genomic signature predicting Trastuzumab-based treatment responses in breast cancer patients, such as the MTORC1 signaling predicting sensitive response, a major downstream effector of HER2 signaling, and the ER and EMT pathways predicting resistant response, both of which are known to endow HER2 therapy and chemotherapy resistance in breast cancer.

In conclusion, iGenSig represents a unique class of white-box methods for big-data based precision oncology with specialized algorithms adapted to the hallmark characteristics of genomic data, and is designed to address the transparency, cross-dataset applicability, and interpretability for big-data based modeling. iGenSig-Rx will have broad applications on modeling therapeutic responses and clinical tumor behaviors based on multi-omics datasets of clinical trials or retrospective clinical studies.

### Conclusions

iGenSig-Rx modeling generates predictive scores using redundant genomic features associated with therapeutic responses found in labeled genomic datasets, term as an integral genomic signature. To mitigate feature redundancy, we adaptively penalize the occurrence of predictive features identified in particular tumors using unlabeled datasets across large tumor cohorts. iGenSig modeling minimize the sequencing bias effects by capturing the integral predictive signal and implementing purpose-built algorithms to address the biological characteristics observed in the data, which are often beyond the detection capability of machine learning models limited by smaller subject sizes.

We utilized genomic datasets from CALGB40601, a neoadjuvant phase III trial employing trastuzumab and paclitaxel-based chemotherapy, to develop the iGenSig-Rx model. This model aimed to predict therapeutic responses among HER2 + breast cancer patients undergoing this treatment regimen. In our evaluation, the iGenSig-Rx model demonstrated strong predictive ability within the CALGB 40601 subjects, showcasing an average AUROC of 0.91 in training sets and 0.89 in internal test sets. For cross-dataset performance assessment, we benchmarked the model against three external validation sets: ACOSOG Z1041, NOAH, and NSABP B41. As anticipated, the iGenSig models exhibited superior performance, achieving an AUROC of 0.80 in the ACOSOG Z1041 trial, which included both gene expression and whole exome sequencing (WXS) data. In contrast, in the NOAH and NSABP B41 trials, where only transcriptomic data was available, the model delivered AUROCs of 0.75 each.

The iGenSig-Rx model showcased remarkable cross-dataset performance and robustness against simulated errors in genomic features. Notably, it surpassed standard machine learning and AI methods in three distinct clinical trials. Crucially, the iGenSig-Rx model presents the much-needed transparency for clinical application. It provides clear insights into the prediction process, elucidating how features contribute to predictions and highlighting the key underlying pathways. Interpreting the iGenSig-Rx model yielded clinically relevant insights into the signature pathways at play.

## Data Availability

The data used in this study are from public databases that require controlled access (see methods). The R modules are available through: https://github.com/wangxlab/iGenSig-Rx (the Github repository will be released upon publication. The R modules and data files are available for review through the following link: https://drive.google.com/drive/folders/1KgecmUoon9-h2Dg1rPCyEGFPOp28Ols3?usp=sharing)
